# Successful Management of a Combined Abdominal and Thoracic Trauma with Rectal Impalement: Report of a Case

**DOI:** 10.1155/2013/816089

**Published:** 2013-06-17

**Authors:** Konstantinos Kasapas, Anna Daskalaki, George Kaimasidis, George Chalkiadakis

**Affiliations:** ^1^Department of General Surgery, Chania General Hospital, 73300 Chania, Greece; ^2^Department of Neurosurgery, Athens General Hospital “G. Gennimatas”, 11527 Athens, Greece; ^3^Department of General Surgery, Heraklion University Hospital, 71110 Heraklion, Greece

## Abstract

*Introduction*. Combined abdominal and thoracic impalement injuries are a rare form of penetrating trauma. Nowadays, they occur more frequently as an accident and not so often as a deliberate violent action. *Case Report*. A 35-year-old man was admitted to our emergency department with chest pain and respiratory distress after he had reportedly slipped in his bathtub. Abdominal and thoracic imaging, including computed tomography (CT), confirmed a right-sided pneumothorax and a liver laceration without bleeding or further endoperitoneal trauma. A chest tube was placed. During his hospitalization in the first 24-hour period, he complained of abdominal and right shoulder pain accompanied by fever. A new abdominal and thoracic CT scanning revealed a rupture of the rectosigmoid, a rupture of right hemidiaphragm, and a foreign body in the thoracic cavity. The patient admitted that a broomstick was violently placed through his rectum, and he underwent a thoracotomy with an exploratory laparotomy. The foreign object was removed, the diaphragmatic rupture was repaired, and a Hartmann's procedure was performed. The postoperative course was uneventful. *Conclusion*. In cases of combined thoracoabdominal trauma, high index of suspicion is required when medical history is misleading and the injuries are not obvious immediately. A coordinated team effort in a well-organized trauma center is also very important.

## 1. Introduction 

Impalement injuries are usually the result of accidental falls onto an object or a car accident but not so often as a result of a torture or execution. We present a rare case of a thoracoabdominal trauma following rectal impalement and the successful management of injuries that were secondarily revealed because of vagueness in medical history.

## 2. Case Report 

**Figure 1:**
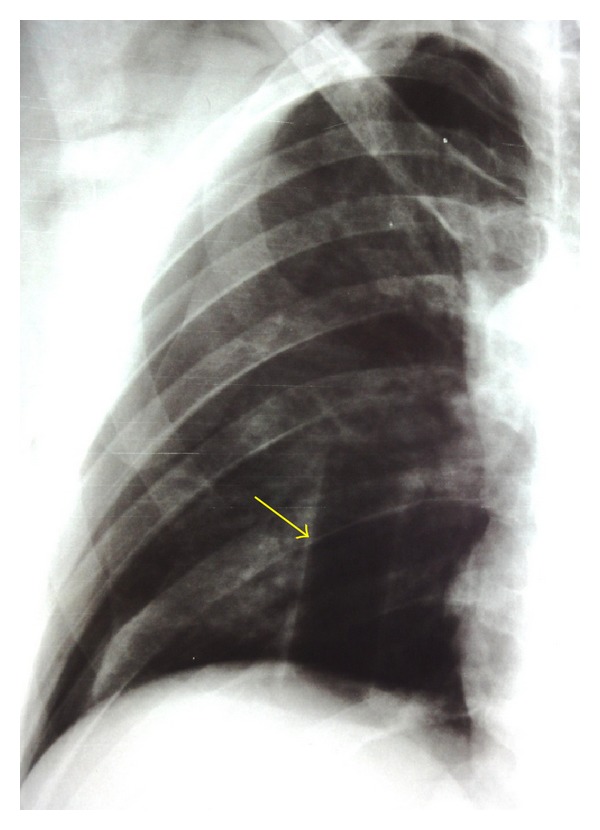
Chest X-ray showing a foreign body into the right thoracic cavity (yellow arrow).

**Figure 2:**
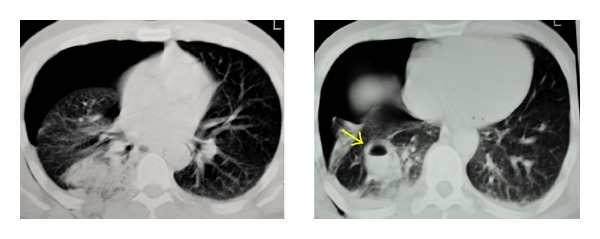
Chest CT scan showing a right-sided pneumothorax and an oval shaped lesion with an air fluid level (foreign body) (yellow arrow).

**Figure 3:**
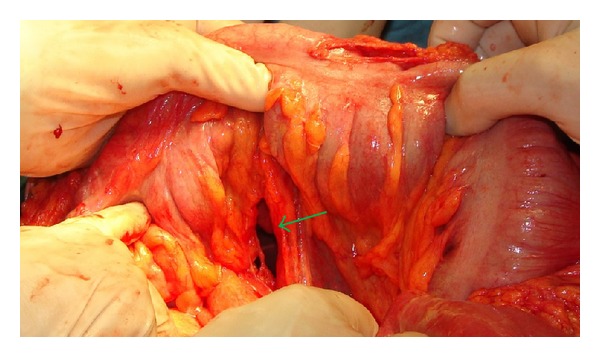
Perioperative recognition of transverse mesocolon rupture (green arrow).

**Figure 4:**
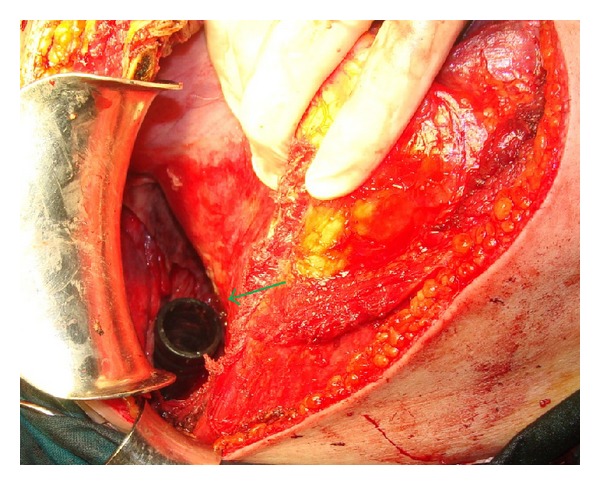
The rounded end of the broomstick in the right thoracic cavity (green arrow).

**Figure 5:**
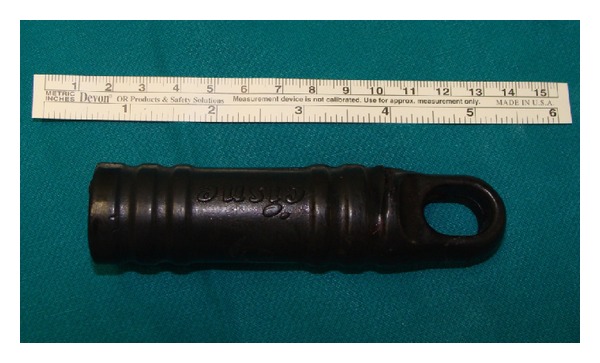
The plastic rounded end of the broom handle after its removal.

A 35-year-old man was admitted to our emergency department with the chief complaint of right hemithoracic pain and respiratory distress. He reportedly slipped in his bathtub. Blood pressure, pulse rate, respiratory frequency, hemoglobin, and blood chemistry findings were all normal. On physical examination, decreased breath sounds on the right side were found. After assurance of the patient's hemodynamic stability, abdominal and thoracic imaging with CT scanning was obtained. A right-sided pneumothorax and a small liver laceration in segment VIII without any active bleeding or further intraperitoneal trauma were found. A chest tube for air removal was placed at that moment. The next day, the patient complained of abdominal pain which radiated to the right shoulder and had a fever of 39°C. A chest X-ray and a new abdominal/thoracic CT scan were obtained and the following findings were detected: rupture of the rectosigmoid, liver laceration in segment VIII, rupture of the right hemidiaphragm, and a foreign body in the thoracic cavity (Figures 1 and 2). On digital examination, the rectal ampulla was found without any blood clots, and laboratory findings were all normal. Repeated questioning led to the eventual discovery of the correct etiology of the trauma—the patient admitted that a broomstick was violently placed through his rectum that its plastic rounded end probably stayed in his body. The patient was brought to the operating room so that his injuries could be explored and operatively managed. Both thoracic surgeons and abdominal surgeons took part. Median sternotomy and exploratory laparotomy were performed. The broom had pierced the rectosigmoid junction and the transverse mesocolon (Figure 3), had lacerated the segment 8 of liver, and had pierced the right diaphragm. In the right pleural cavity, an empyema and the rounded end of the broomstick were also discovered (Figure 4). There were not any vascular injuries. The foreign object was removed (Figure 5), and a lung decortication was done with simultaneous removal of pseudomembranes and pus. The diaphragmatic laceration was then repaired. Due to high suspicion of intraperitoneal sepsis because of peritonitis, a Hartmann's procedure with end colostomy was performed. Thoracostomy tubes were bilaterally placed and an intra-abdominal drainage as well. Postoperatively, the patient was treated with antibiotics and stayed in the intensive care unit for four uneventful days. He was hospitalized for 25 days and was discharged in good condition with a temporary colostomy.

## 3. Discussion

Impalement injuries are caused by penetration of elongated objects through the body. These injuries usually result from accidental falls onto stable objects, car accidents, or, less commonly, violent actions. Penetrating impalement injuries usually involve a through-and-through mechanism. They are often multiorgan depending mostly on the entry site, the direction, and the nature of the penetrating object. 

As far as anorectal impalement injuries are concerned, they usually result from penetrating traumas (gunshot and stab wounds) or, less commonly, from transanal injuries [1]. Transanal injuries include iatrogenic cases, sexually related activities [2], or violent actions. These injuries are sometimes lethal [3]. The characteristics of these last two types of injury are the delay in seeking medical assistance and the vagueness of the medical history. Sometimes the real cause is not admitted due to social reasons. A careful approach to children with this type of injury is necessary because sexual abuse is the major cause of rectal injuries, and a medical doctor should always think of this possibility [4].

When the impaling object is still visible on admission, the clinical situation is clearer. However, in concealed impalement injuries with an unclear medical history, as was this case, clinical attention must be paid because part of the object may still hide inside the body, possibly without early symptoms or signs [5]. 

Upon evaluation of patients with penetrating trauma, the potential for diaphragmatic involvement and organ injuries in both the thoracic and abdominal cavities must always be suspected. Diaphragmatic rupture mostly occurs in penetrating rather than blunt trauma [6–9]. The traumatic diaphragmatic rupture can easily be missed due to the severity of coexisting injuries and its silent nature in many cases. Among the patients with thoracoabdominal penetrating trauma, the diagnosis is easier in those patients that undergo a laparotomy for injuries in the abdomen. Nevertheless, at the time of surgery, some diaphragmatic injuries can be overlooked, so it is recommended to examine thoroughly both hemidiaphragms during the operation [8, 9]. Beigi et al. report that the true incidence of diaphragmatic injury is unknown because in 7–66% of major trauma victims, the diagnosis is missed. Chest X-ray and CT scan are diagnostic in only 33.3% of cases [8].

Rectal impalement is a rare injury, and specialized knowledge or experience on the management of this type of injury is sparse. Review of the English literature revealed the existence of three other cases with a similar mechanism (object traversing from pelvic to thoracic cavity) [10–12]. Our case is of particular interest due to the survival of the patient from a potentially lethal trauma that involved many organs. The medical history was misleading, and the remaining object was finally observed and removed because of the 24-hour close observation of the patient, although it was not revealed at first evaluation.

## 4. Conclusion 

Combined thoracic and abdominal trauma after rectal impalement is a serious medical situation which demands cooperative effort from different medical subspecialties. In our case, both abdominal and cardiothoracic surgeons took part in the management of the patient. It is also crucially important for a trauma surgeon to be suspicious of associated injuries and to have the patient under at least a 24-hour close observation especially when there is a suspicion of a misleading medical history. The trauma of the visceral organs is often multiple, and the management can be altered according to the needs of the patient.
